# The Behavioral Effects of tDCS on Visual Search Performance Are Not Influenced by the Location of the Reference Electrode

**DOI:** 10.3389/fnins.2017.00520

**Published:** 2017-09-21

**Authors:** Amanda Ellison, Keira L. Ball, Alison R. Lane

**Affiliations:** ^1^Cognitive Neuroscience Research Unit, Department of Psychology, Durham University Durham, United Kingdom; ^2^Wolfson Research Institute for Health and Wellbeing, Durham University Durham, United Kingdom

**Keywords:** tDCS, right PPC, right FEF, montage, reference electrode, visual search

## Abstract

We investigated the role of reference electrode placement (ipsilateral v contralateral frontal pole) on conjunction visual search task performance when the transcranial direct current stimulation (tDCS) cathode is placed over right posterior parietal cortex (rPPC) and over right frontal eye fields (rFEF), both of which have been shown to be causally involved in the processing of this task using TMS. This resulted in four experimental manipulations in which sham tDCS was applied in week one followed by active tDCS the following week. Another group received sham stimulation in both sessions to investigate practice effects over 1 week in this task. Results show that there is no difference between effects seen when the anode is placed ipsi or contralaterally. Cathodal stimulation of rPPC increased search times straight after stimulation similarly for ipsi and contralateral references. This finding does not extend to rFEF stimulation. However, for both sites and both montages, practice effects as seen in the sham/sham condition were negated. This can be taken as evidence that for this task, reference placement on either frontal pole is not important, but also that care needs to be taken when contextualizing tDCS “effects” that may not be immediately apparent particularly in between-participant designs.

## Introduction

Transcranial direct current stimulation (tDCS) involves passing an electrical current from an anode to a cathode (Been et al., 2007; Sparing and Mottaghy, 2008) resulting in a change in neuronal excitability. Anodal stimulation increases the likelihood of neuronal firing under that electrode, widely believed to lead to improvements in task performance (Kincses et al., 2003; Nitsche et al., 2003c; Fregni et al., 2005; Bolognini et al., 2010). Conversely, cathodal stimulation reduces neuronal firing (Nitsche and Paulus, 2000; Antal et al., 2006; Been et al., 2007) and is associated with deficits in performance or increasing thresholds for phosphene detection by reducing excitability (Nitsche and Paulus, 2000, 2001; Antal et al., 2003; Nitsche et al., 2003a; Berryhill et al., 2010).

The use of tDCS to understand the neural function in cognitive tasks typically however takes a bipolar non-balanced approach (Nasseri et al., 2015), investigating the region of interest over which the cathode or anode is placed. The complementary electrode is termed simply the reference electrode and is placed most often over the contralateral frontal pole (Nitsche et al., 2008; Stagg and Nitsche, 2011). This approach, at best denigrates, or at worst ignores, the possible involvement that modulation of the underlying frontal regions may have in the processing of the cognitive task at hand. Furthermore, modulation of activity in this region may contribute to the detriment/improvement in function following stimulation. In the experiment reported in this paper, we sought to evaluate the role of the reference electrode in a task which has been extensively investigated with respect to neurostimulation and underpinning functional networks.

Imaging studies have shown that the effects of tDCS are not limited to the sites of the two electrodes but rather widespread brain regions are modulated (Lang et al., 2005; Priori et al., 2009; Polanía et al., 2010; Pena-Gomez et al., 2011; Ellison et al., 2014), and given the number of factors influencing neuronal modulation, computer models have been critical to understanding the patterns of neuronal firing following tDCS (Bikson and Datta, 2012). Ranging in complexity these models take into account both anatomical differences (for example, Sadleir et al., 2010) and experimenter defined stimulation parameters (Wagner et al., 2007; Datta et al., 2008). While maximum current is discharged directly below the electrodes (Bai et al., 2012) the placement of both the active and reference electrodes affects neuromodulation under the active electrode (Bikson et al., 2010).

While computer models have been invaluable in directing stimulation practices and allowing predictions to be made about the regions of effect for different electrode placements, and electrode montage categories (for example, Nasseri et al., 2015) it is not clear what the consequences of different electrode placements are on behavior. One study evaluating the behavioral consequences of electrode placement on MEP thresholds, found a negative correlation between the distance between the two electrodes and the degree of neuronal modulation (Moliadze et al., 2010), mirroring model predictions (Datta et al., 2008, 2009). However, data looking at the effect of electrode placement on cognitive behavior are lacking. Here we sought to evaluate the behavioral consequences of cathodal stimulation in both a unilateral-bipolar montage and a bilateral bipolar-nonbalanced montage. Two regions of interest were investigated (right frontal eye fields [rFEF] and right posterior parietal cortex [rPPC]) in one behavioral task (conjunction visual search).

Both the frontal eye fields (FEFs) and the right posterior parietal cortex (rPPC) are involved in visual search as shown by imaging studies (Corbetta et al., 1991; Corbetta and Shulman, 1998; Donner et al., 2002). Furthermore, transcranial magnetic stimulation (TMS) studies have demonstrated that these areas have a causal involvement in visual search, particularly in conjunction searches (Ashbridge et al., 1997; Ellison et al., 2003; Muggleton et al., 2003; O'Shea et al., 2006; Anderson et al., 2007; Lane et al., 2011, 2012). We have previously demonstrated (using a contralateral frontal pole electrode) that while the effects of anodal stimulation to both rPPC and rFEF are no different than those in a sham stimulation condition, the application of cathodal stimulation to these two areas resulted in different effects (Ball et al., 2013). When applied to rPPC, cathodal stimulation resulted in a slowing in search times for trials completed when the stimulation was being applied and the typical search time benefit of practice over subsequent blocks was not observed after the stimulation had ceased. Conversely, when cathodal stimulation was applied to rFEF search times were no different to sham search times.

Given these observations, the current study is restricted to cathodal stimulation only.

For both sites, the anode was either above the participant's left supraorbital cortex (contralateral to the cathode) or above the participant's right supraorbital cortex (ipsilateral to the cathode) to allow us to investigate whether search behavior is influenced by the position of the reference electrode. Placing the reference electrode over the supraorbital cortices was chosen due to the prevalence of this montage in the current transcranial electrical stimulation literature (see Stagg and Nitsche, 2011).

## Methods

### Participants

Thirty five participants (14 male) took part in this experiment took part in this experiment (age range 18–41 years, mean age 23.7, *SD* = 5.7, 27 right handers). Participants were randomly assigned to one of five conditions. All participants were from Durham University and had normal or corrected-to-normal vision. Participant selection complied with the current guidelines for repetitive tDCS research and this study was carried out in accordance with the recommendations of Durham University Ethics committee with written informed consent from all participants. All participants gave written informed consent in accordance with the Declaration of Helsinki and with the approval of Durham University Ethics Advisory Committee.

### Stimuli presentation

The experiment was run on an IBM compatible personal computer with a 16-inch monitor (1,024 by 768 resolution, refresh rate 60 Hz) and was programmed using E-prime (Psychology Software Tools Inc., Pittsburgh, PA, USA). The viewing distance was 57 cm and the center of the screen was at eye level, with a chin rest being used to ensure that this was maintained. The experiment was completed in a dark room.

### Visual search task

**Figure 1:**
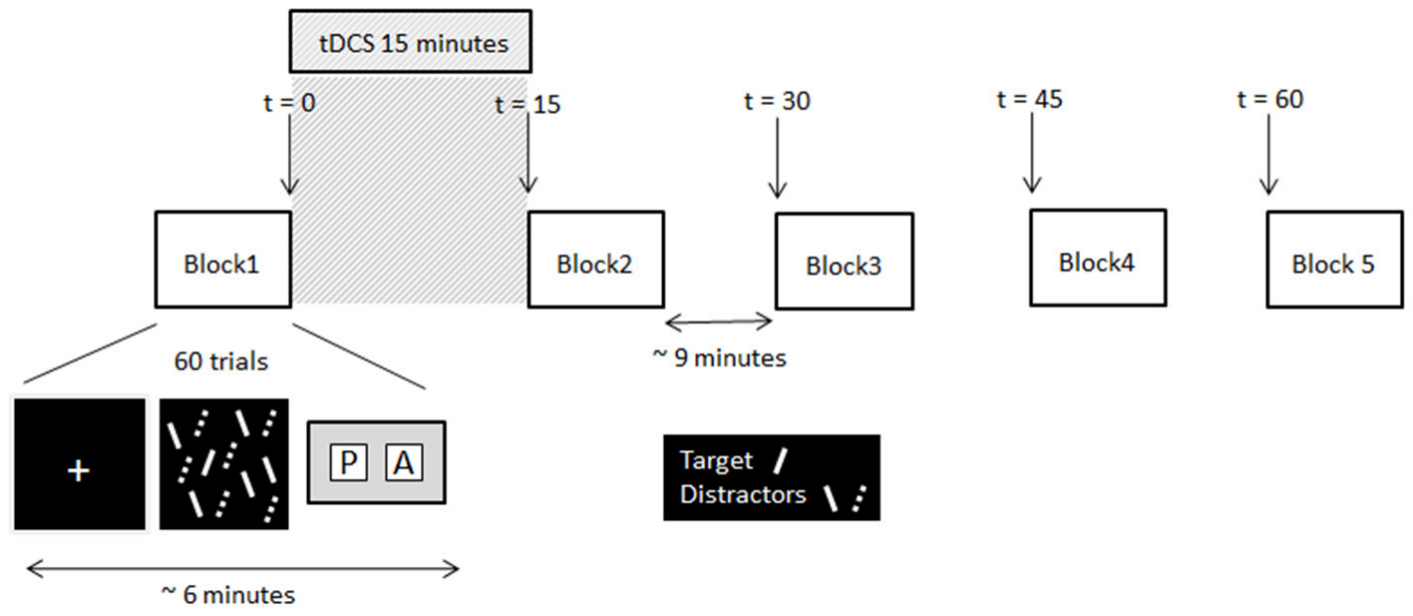
Schematic of the experimental procedure and timing information. Note, the solid lines and dashed lines were solid red and solid green lines respectively in the displays presented to participants.

The search arrays consisted of red and green lines on a black background (Figure 1). The target was always a red forward slash (oriented at 45° from vertical) and distractors were green forward slashes and red backslashes (oriented at −45° from vertical). Search arrays contained 12 items: in target present trials there was one target and 11 distractors (five red backslashes and six green forward slashes), and in target absent trials there were 12 distractors (six red backslashes and six green forward slashes). The target was present on 50% of trials and appeared on the left and right side of the array equally. Each line measured 2.5° of visual angle in length and 0.4° of visual angle in width. The whole screen measured 32° of visual angle horizontally and 24° vertically. The 12 items in each search array were randomly placed into a 10 × 6 virtual grid to prevent items overlapping.

### Procedure

At the beginning of each trial a white fixation cross (0.5° of visual angle) was presented centrally for 500 ms. This was followed immediately by the search array. Participants had to decide as quickly and as accurately as possible whether the target was present or absent, and make the corresponding key-press response (Cedrus RB-620 button box, San Pedro, California). The search array remained on the screen until the participant responded. A blank screen was then presented for a variable duration (from 3,000 to 5,000 ms) before the next trial was initiated. No feedback about whether the correct response was made was given. Participants completed two testing sessions, separated by 1 week. In each session participants completed five blocks of visual search trials (30 target present and 30 target absent trials randomly presented per block). Each block took approximately 6 min to complete. Upon completion of the first block of the session participants received 15 min of tDCS (or sham stimulation depending on session), after which they completed the remaining four blocks of trials at 15 min intervals. The timeline of the experiment is illustrated in Figure 1. In the time between blocks participants sat quietly until they were instructed to start the next block. All participants received Sham stimulation in week 1, providing each participant with their own baseline to which their search times after tDCS were compared.

### Transcranial direct current stimulation

The two rubber electrodes were placed in two sponge pouches (7 × 5 cm) which had been soaking in a physiologically active saline solution. A rubber strap was used to hold the two electrodes in place. tDCS was applied using a Magstim Eldith DC stimulator for 15 min at a current intensity of 1.0 mA. This level of stimulation was selected given previous reports that 1.0 mA is sufficient at inducing measureable changes in performance (Rogalewski et al., 2004; Fregni et al., 2006; Stagg et al., 2009). Stimulation protocol complied with the current safety guidelines for tDCS (Nitsche et al., 2003b). In the second testing session, participants in all but the sham condition, received 15 minutes of stimulation, whereby the cathode (active) electrode was placed over either the right posterior parietal cortex (rPPC) or the right frontal eye field (rFEF). The anode (reference) electrode was placed either above the participant's left supraorbital cortex (contralateral to the cathode) or above the participant's right supraorbital cortex (ipsilateral to the cathode). In the first testing session all participants received stimulation for only 30 s; consequently they experienced the initial itching sensation associated with real stimulation but insufficient current for any neuronal modulation; therefore, participants were not aware which stimulation condition they were experiencing in each week. A between-groups design was used whereby participants only completed one condition: rPPC with an ipsilateral reference, rPPC with a contralateral reference, rFEF with an ipsilateral reference, rFEF with a contralateral reference, or sham stimulation (Sham) whereby participants received sham stimulation in both sessions.

**Figure 2:**
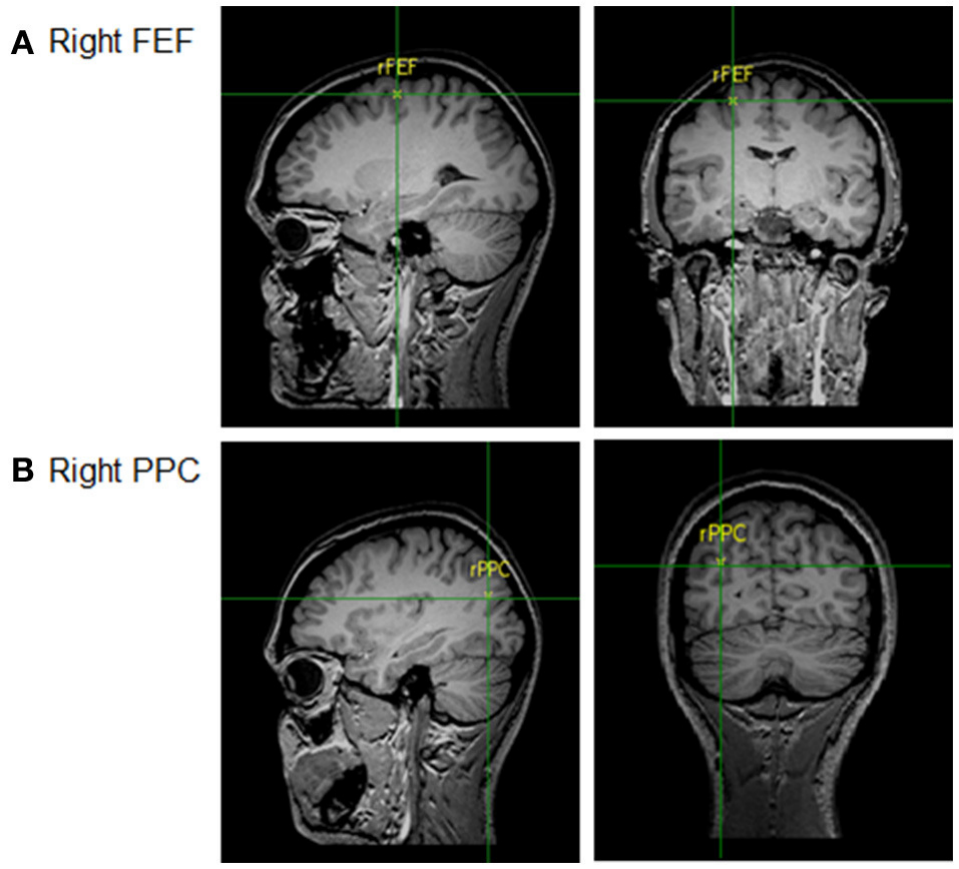
Locations of tDCS sites: **(A)** Right FEF and **(B)** Right PPC.

The rPPC was measured as being 9 cm dorsal and 6 cm lateral to the right of the mastoid-inion as this corresponds with the angular gyrus known for its role in visual search tasks using TMS (Ashbridge et al., 1997; Ellison and Cowey, 2009). The rFEF site was located at 5 cm lateral toward the right and 4 cm anterior from the vertex, corresponding with the confluence of the right pre-central gyrus and right superior frontal gyrus, the location of rFEF (Paus, 1996). The locations of the two brain sites are shown in Figure 2. The area of stimulation was defined by the size of the electrodes (Peterchev et al., 2011), thus, precise functional localization of the sites of interest was not necessary and centring the electrode over the known regions was sufficient.

## Results

### Data analysis

Analyses were carried out on reaction times for target present trials only. Participants were correct on 95.9% of target present trial and this was not significantly different between testing sessions [Session 1: *M* = 95.3%; Session 2: *M* = 96.4%, *t*_(34)_ = −1.898, *p* = 0.066] Responses to incorrect trials were removed, as were search times more than two standard deviations above or below the individual's mean (9.6% of correct present trials were excluded on these grounds). All data were tested for normality using the Shapiro–Wilk statistic; the data were normal unless otherwise stated. Inferential statistics used a significance level of *p* < 0.05, except when multiple comparisons were performed, when a Bonferonni correction was applied.

#### Global analysis

A mixed model ANOVA with the between group factors of Site (rFEF, rPPC) and Reference Placement (Contralateral, Ipsilateral) and the within group factors of Block (1–5) and Session (1, 2) was performed (this analysis did not include the data from the participants who “received” sham stimulation in both sessions). The analysis revealed significant main effects of Session, *F*_(1, 24)_ = 30.12; *p* < 0.05, and Block, *F*_(4, 96)_ = 5.29; *p* < 0.05. The interaction between Session and Block was also significant, *F*_(4, 96)_ = 6.17; *p* < 0.05. As all participants received sham stimulation in session 1 and cathodal tDCS in session 2 this interaction suggests that search times in the second session are being modulated by either the effect of practice or by the application of tDCS. The main effect of Site was not statistically significant (*p* = 0.441), and of interest the main effect of Reference Placement (*p* = 0.144), along with all other interactions, were not significant.

To investigate the Session x Block interaction, an analysis regarding the equivalence of sham reaction times across groups was first performed, followed by separate analyses for each stimulation condition.

#### Session 1 search times

Each participant completed two testing sessions with all participants receiving sham stimulation in the first session, thus each participant has their own sham data enabling within participant comparisons to be made. To ensure that the sham data across the five participant groups are equivalent an ANOVA with the within groups factor of Block (1–5) and the between group factor of Stimulation Condition (rPPC Ipsilateral, rPPC Contralateral, rFEF Ipsilateral, rFEF Contralateral, and Sham) was performed on search times from the first testing session only. The analysis revealed a significant main effect of Block, *F*_(4, 120)_ = 10.33; *p* < 0.05, a non-significant main effect of Stimulation Condition (*p* = 0.323), and a non-significant interaction between Block and Stimulation Condition (*p* = 0.311). Therefore, we can be confident that the Sham data for each condition are not significantly different from each other. Furthermore, a one-factor ANOVA for the five stimulation conditions found no difference in search times at Block 1 (*p* = 0.164).

#### Sham stimulation in both sessions

**Figure 3:**
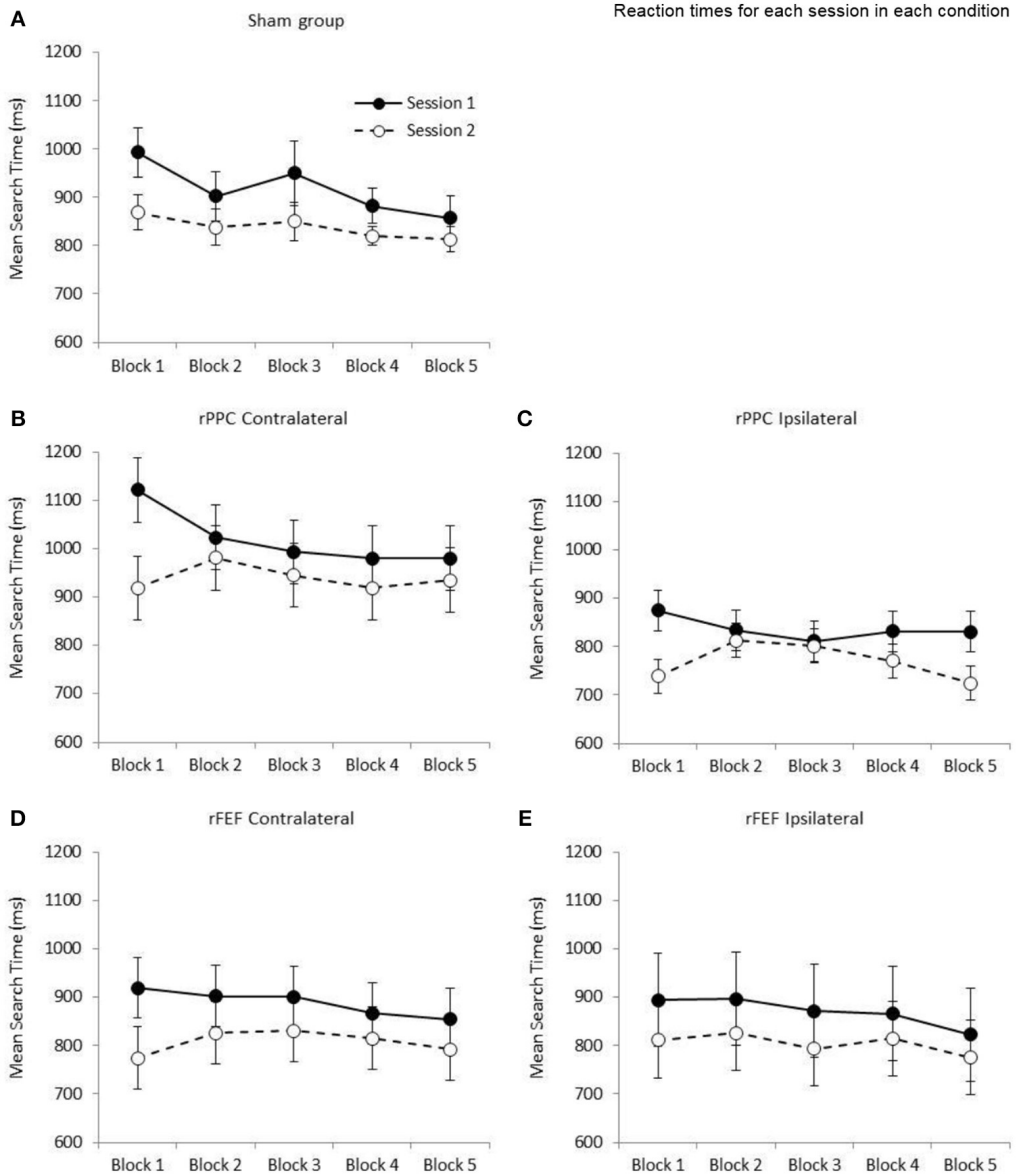
Graphs showing the mean search times (ms) for the five stimulation conditions: **(A)** Sham group; **(B)** cathode over rPPC and a contralateral reference; **(C)** cathode over rPPC and an ipsilateral reference; **(D)** cathode over rFEF and a contralateral reference; **(E)** cathode over rFEF and an ipsilateral reference. The solid lines and filled circles represent session 1 data (all participants received sham stimulation in this session). The dashed lines and open circles represent session 2 data. A one factor ANOVA found no difference between the five stimulation conditions at the first block in Session 2 (*p* = 0.235). Error bars represent ±1 standard error of the mean for each condition.

Analyzing only the search times of those participants who received sham stimulation in both sessions allows us to separate the effects of practice and the effects of tDCS on search times. This data is shown in Figure 3A. A 2 × 5 repeated measures ANOVA with the factors of Session (1, 2) and Block (1–5) revealed a significant main effect of Session, *F*_(1, 6)_ = 7.31; *p* < 0.05, such that search times were slower in Session 1 (*M* = 916.59, *SE* = 47.2) compared to Session 2 (*M* = 837.55, *SE* = 25.8). While the main effect of Block was also significant, *F*_(4, 24)_ = 5.35; *p* < 0.05, the interaction between Session and Block was not statistically significant (*p* = 0.317). To provide an overall measure of search performance search time comparisons are made between the first and fifth blocks of each session. In both sessions participants became significantly faster between the first and fifth blocks of trials [Session 1: *t*_(6)_ = 3.77; *p* = 0.009, mean reduction of 135.83 ms, *SE* = 36.1, *r*^2^ = 0.703; Session 2: *t*_(6)_ = 3.17; *p* = 0.019, mean reduction of 55.53 ms, *SE* = 17.5, *r*^2^ = 0.626]. Given that a reduction in search times, albeit a smaller reduction, was observed in the second session, which we credit to further practice with the task, we compare search times for each site and electrode montage separately.

#### Stimulation of rPPC with a contralateral reference electrode

The ANOVA with the factors of Session (1, 2) and Block (1–5) revealed a significant main effect of Session, *F*_(1, 6)_ = 13.6; *p* < 0.05, such that search times were slower in Session 1 (*M* = 1,019.97, *SE* = 60.4) compared to Session 2 (*M* = 940.13, *SE* = 63.8). While the main effect of Block was not significant (*p* = 0.077), the Session by Block interaction was significant, *F*_(4, 24)_ = 3.31; *p* < 0.05, indicating that search performance was different across the two sessions (see Figure 3B). Comparing search times between the first and fifth blocks, while participants became significantly faster across the five blocks in Session 1, *t*_(6)_ = 2.77; *p* = 0.032, *r*^2^ = 0.561, with a mean reduction of 141.14 ms (*SE* = 51.1), in Session 2 search times did not change (*p* = 0.654, increase of 15.97 ms, *SE* = 33.9). Based on the data of participants who received sham stimulation in both sessions (presented in Figure 3A) a reduction in search times was expected across the five blocks of session 2; therefore, it appears that the application of cathodal tDCS to rPPC with a contralateral reference electrode is negating the practice effect. tDCS was applied after the first block of the second session and the slowing in search times between blocks 1 and 2 of the second session is statistically significant, *t*_(6)_ = 4.28; *p* < 0.05 (mean slowing of 61.82 ms, *SE* = 14.5). This is seen in Figure 3B.

#### Stimulation of rPPC with an ipsilateral reference electrode

The ANOVA with the factors of Session (1, 2) and Block (1–5) revealed a significant main effect of Session, *F*_(1, 6)_ = 7.47; *p* < 0.05, such that search times were slower in Session 1 (*M* = 836.52, *SE* = 38.9) compared to Session 2 (*M* = 769.57, *SE* = 32.2). While the main effect of Block was not significant (*p* = 0.126), the Session by Block interaction was again significant, *F*_(4, 24)_ = 4.06; *p* < 0.05. Comparing search times between the first and fifth blocks, while participants became significantly faster across the five blocks in Session 1, *t*_(6)_ = 2.53; *p* = 0.045, *r*^2^ = 0.516 with a mean reduction of 43.97 ms (*SE* = 17.4), in Session 2 search times did not change between the first and fifth blocks (*p* = 0.659, reduction of 14.56 ms, *SE* = 31.4). Again, it would appear that cathodal tDCS is negating the expected speeding in search times (see Figure 3C). Similar to the rPPC contralateral data, there was a significant slowing of search time between the first two blocks of the second session, *t*_(6)_ = 2.85; *p* < 05, *r*^2^ = 0.575 (*M* = 73.10 ms, *SE* = 25.6).

#### Stimulation of rFEF with a contralateral reference electrode

The ANOVA with the factors of Session (1, 2) and Block (1–5) revealed a significant main effect of Session, *F*_(1, 6)_ = 7.14; *p* < 0.05, such that search times were slower in Session 1 (*M* = 888.91, *SE* = 60.1) compared to Session 2 (*M* = 807.70, *SE* = 63.2). The main effect of Block was not significant (*p* = 0.165), and likewise the Session by Block interaction (*p* = 0.240). Comparing search times between the first and fifth blocks of a session, while participants became faster across the five blocks in Session 1, *t*_(6)_ = 2.45; *p* = 0.050 (which does not survive correction for multiple comparisons) with a mean reduction of 64.55 ms (*SE* = 26.3, *r*^2^ = 0.500), in Session 2 search times were no different between Blocks 1 and 5 (*p* = 0.332, increase of 18.90 ms, *SE* = 17.9, see Figure 3D). There was no significant difference in search times between the first two blocks of the second session (*p* = 0.088, *M* = 52.61 ms, *SE* = 25.9).

#### Stimulation of rFEF with an ipsilateral reference electrode

The ANOVA with the factors of Session (1, 2) and Block (1–5) revealed a marginally non-significant main effect of Session (*p* = 0.068, with search times slower in Session 1: *M* = 870.21, *SE* = 96.7; compared to the Session 2: *M* = 804.34, *SE* = 74.3). The main effect of Block was non-significant (*p* = 0.290), and likewise there was non-significant Session by Block interaction (*p* = 0.847). Participants were faster in the fifth block of Session 1 with respect to the first with a mean reduction of 71.65 ms (*SE* = 35.6) however this did not reach significance (*p* = 0.091). The comparable difference in Session 2 was 35.52 (*SE* = 44.8, *p* = 0.458, see Figure 3E). There was no significant difference in search times between the first two blocks of the second session (*p* = 0.652, *M* = 15.23 ms, *SE* = 32.1).

#### Comparing electrode placements: normalized effects—immediate tDCS effects

**Figure 4:**
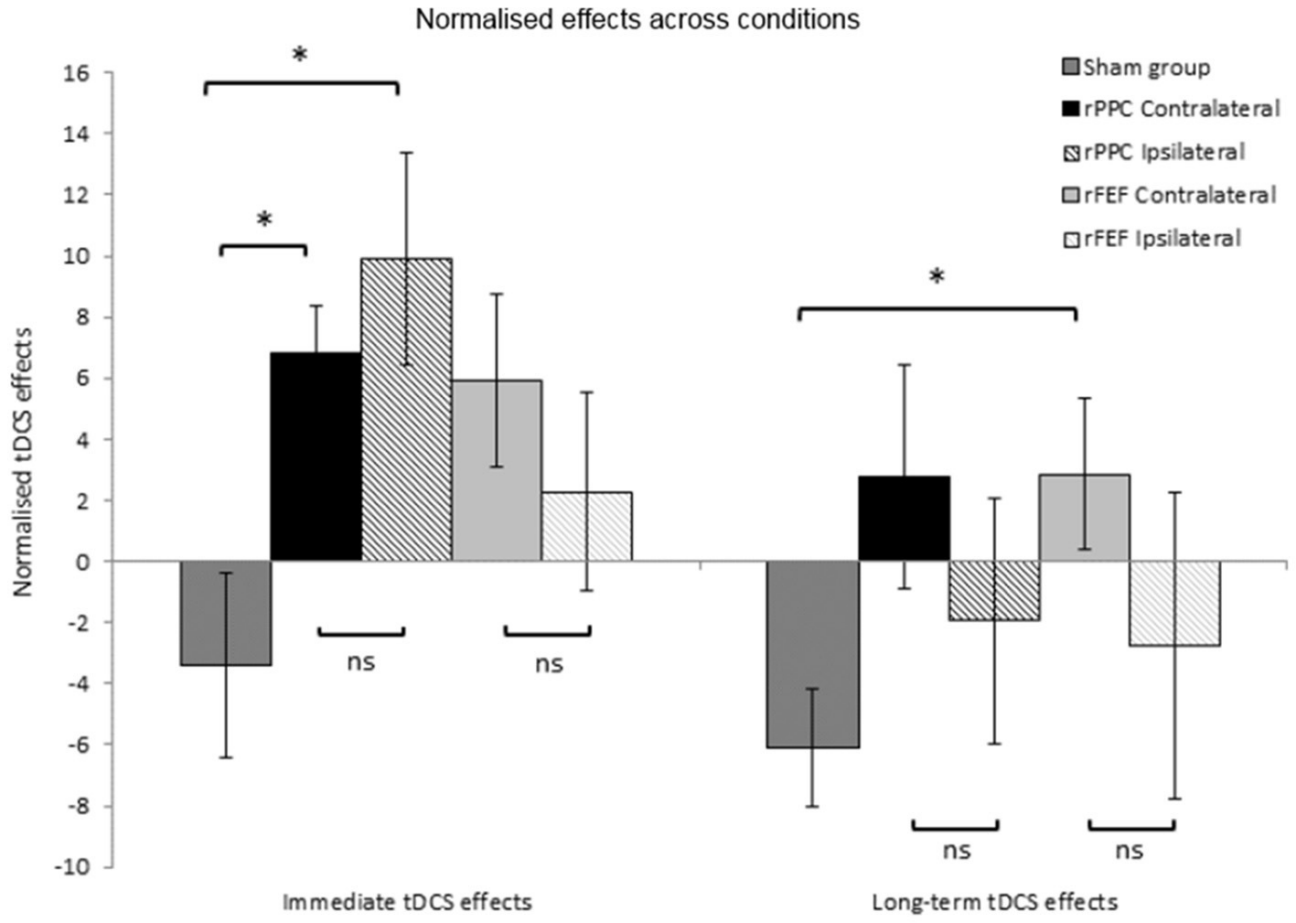
Normalized tDCS effects expressed as a percentage of search times to Block 1 of session 2 (the block prior to the application of tDCS). Immediate tDCS effects compare search times between blocks 1 and 2 and long-term tDCS effects compare search times between blocks 1 and 5. A positive number indicates slower search times following tDCS. ^*^p < 0.05. Error bars represent ± 1 standard error of the mean for each condition.

With the exception of the participants who received Sham stimulation in both sessions, all other participants received tDCS in the second session. The first block of trials of the second session was completed before the stimulation; therefore, providing a within session baseline of search performance, and the second block of trials of this session was completed immediately after the stimulation had ceased. Comparing search times for these two blocks of trials provide a measure of the immediate within-participant effects of tDCS. We calculated the percentage change in search times between these two blocks to provide a normalized immediate tDCS effect allowing between group comparisons to be made. For each participant the following equation was used: (Block 2 search time—Block 1 search time)/(Block 1 search time) × 100/1, with a positive number indicating that search times became slower between Blocks 1 and 2. The mean normalized immediate tDCS effect was then calculated for each group of participants. Independent samples *t*-tests found no difference between the immediate tDCS effects for the two rPPC groups (Contralateral: *M* = 6.83, *SE* = 1.6, Ipsilateral: *M* = 9.92, *SE* = 3.4, *p* = 0.431), or between the two rFEF groups (Contralateral: *M* = 5.93, *SE* = 2.8, Ipsilateral: *M* = 2.28, *SE* = 3.3, *p* = 0.414, see left hand panel of Figure 4). This further suggests that the tDCS effects were not modulated by reference electrode placement.

There was a significant difference between the normalized immediate tDCS effects in the rPPC Ipsilateral and Sham groups (Sham: *M* = −3.40, *SE* = 3.0, *t*_(12)_ = 2.90; *p* = 0.013, Cohen's d: 1.55), and likewise between the rPPC Contralateral and Sham groups, *t*_(12)_ = 3.00; *p* = 0.011, Cohen's d: 1.602. There was no difference between the normalized immediate tDCS effects in the rFEF Ipsilateral and Sham groups (*p* = 0.225), and between the rFEF Contralateral and Sham groups [*t*_(12)_ = 2.25; *p* = 0.044, not significant when correcting for multiple comparisons].

#### Comparing electrode placements: normalized effects—long-term tDCS effects

The percentage change in search times between the first and fifth blocks of session 2 was calculated to provide with a normalized long-term tDCS effect. The following equation was used for each participant (Block 5 search time—Block 1 search time)/(Block 1 search time) × 100/1, with a positive number indicating that search times became slower between Blocks 1 and 5. Independent samples *t*-tests found no difference between the long-term tDCS effects for the two rPPC groups (*p* = 0.403), or between the two rFEF groups (*p* = 0.336, see right hand panel of Figure 4).

There was no difference between the long-term tDCS effects in the rPPC Ipsilateral and Sham groups (*p* = 0.369), and likewise between the rPPC Contralateral and Sham groups (*p* = 0.054), rFEF Ipsilateral and Sham groups showed no significant difference between the immediate tDCS effects (*p* = 0.543); however, between the rFEF Contralateral and Sham groups, there was a significant difference in the magnitude of the normalized long-term tDCS effects [*t*_(12)_ = 2.840, *p* = 0.015, Cohen's d: 2.213].

## Discussion

Our primary question related to whether or not the placement of the reference electrode, which in this case was the anode, on either the ipsilateral or contralateral frontal pole, would modulate task performance when the cathode was placed either over rFEF or rPPC, two sites known to be causally involved in conjunction visual search (Ellison et al., 2003; Kalla et al., 2008). Whilst tDCS only had an immediate effect in the cathodal rPPC condition, the effect was similar when the reference (anode) was placed ipsilaterally and contralaterally. Previously we reported the same slowing in search times when participants were performing the search task with concurrent cathodal rPPC stimulation (Ball et al., 2013); however, here the same effects were observed for searches completed immediately after the stimulation period. This demonstrates that the effect of stimulation is robust to online and offline effects (see Stagg and Nitsche, 2011) in addition to the reference montage.

However, in each condition (site or montage), tDCS negated any decreases in search time associated with practice. As tDCS can modulate neuronal firing patterns for up to 90 minutes after the end of the stimulation period (Nitsche and Paulus, 2001; Nitsche et al., 2003a) participants completed four blocks of trials at 15 min intervals after stimulation ceased. For both electrode montages, search times were no different between the block of trials preceding stimulation and the block of trials that started 45 min after the end of stimulation. Whilst on the face of it this lack of a change in search times before and after stimulation appears to suggest that cathodal stimulation had no lasting behavioral effect on search performance, it is necessary to consider the task we used in greater detail. Participants completed two testing sessions, each consisting of five blocks of trials, and cathodal stimulation was applied in the second session only. While search times decreased significantly across the five blocks of the first session for both the contralateral and ipsilateral placements, search times were no different across the five blocks in the second testing sessions. Based on the data we collected from participants who did not receive any stimulation in either session, a speeding in search times was expected in both sessions. Therefore, while it appears that cathodal stimulation is not having a behavioral effect in the second session, it is actually the case that the stimulation is negating the practice effect in the second testing session, replicating previous findings (Ball et al., 2013).

Previously, we observed that for cathodal stimulation to rFEF across the four blocks of the testing session search times became speeded (Ball et al., 2013), thus the finding here that search times did not change suggesting tDCS negated the excepted benefit of practice on search times, is contrary to our previous data. One notable difference between the two studies is that here participants completed two testing sessions with tDCS being applied in the second session only, while in the Ball et al. (2013) study, participants completed only one testing session. Practice with a visual search task will lead to an improvement in performance, typically observable by a reduction in search times, and with practice slow serial searches can become less laborious and become parallel searches in nature (Ahissar and Hochstein, 1997; Ellison and Walsh, 1998). It is possible that over the course of one testing session of visual search trials the benefits of practice on search times are more powerful than any effect of tDCS on search times, hence why we saw no effect of tDCS in our previous one session experiment compared to the current two session experiment. Data from the sham-sham participant group here demonstrates that the greatest benefits are seen in the first session where search times became on average 136 ms faster between the first and fifth blocks of the session, compared to a more modest, although still statistically significant, reduction of 56 ms across the second session. It is increasingly apparent that a thorough understanding of the cognitive task being investigated is critical to evaluating the apparent varying effects of tDCS, especially for cathodal stimulation; for example, the apparent beneficial effect of cathodal stimulation stemming from filtering out noise from distracting stimuli and thus leading to an improvement in task performance (Weiss and Lavidor, 2012) and likewise here while at first glance it would appear cathodal stimulation is having no effect, this is not actually the case. Our theory of the behavioral effects of within- vs. between-participant designs should be however replicated with a larger sample size.

While clearly the route of the current is different for ipsilateral and contralateral reference electrode placement this had no effect on search behavior. Such counterbalancing of reference electrode provides an elegant control for any possible effects anodal stimulation of the frontal pole may be having in a visual search task. We can now say that changes in reaction time behavior seen following cathodal stimulation of rFEF and rPPC in a conjunction visual search task are related to activity in these regions, and not ancillary modulation of the left frontal pole. As the same effects were seen when the anode (reference) was placed over the left and right frontal poles, it is reasonable to assume that these regions are not involved in the processing of the visual search task to the point where increasing the likelihood that these neurons will fire will affect behavior in this task. Equally, it may mean that both areas have the same involvement. We believe there is more evidence to suggest the former is the more parsimonious explanation (see Nobre et al., 2003; Ellison et al., 2014). Our fMRI study which employed cathodal tDCS with contralateral pole reference placement before scanning the participant to investigate activations during the same visual search task showed widespread distal decreases in frontal activation only some of which might be related to the reference electrode (Ellison et al., 2014). It would therefore be important to replicate this and extend to ipsilateral placement of the reference to investigate differences in activity that may belie this apparently similar behavioral consequence bearing in mind the correlative nature of these activations.

As shown by Ellison et al. (2014), multi-technical approaches can been used to excellent effect to manage the correlation problem. We are now at a stage when the neuronal processes that underpin various aspects of visuospatial performance, from perception (Tseng et al., 2012) through to response selection (Yu et al., 2015) and inhibition (Liang et al., 2014) and visuospactial working memory (Hsu et al., 2014) can be elucidated using concurrent tDCS and EEG (employing multiscale entropy analyses) or tDCS and fMRI (for review see Juan et al., 2017). Only by modulating neural activity and recording changes directly in the brain may we begin to understand how behavior is modulated.

## Conclusion

In answer to our research question of whether there is there a difference between ipsilateral and contralateral reference electrode placements, our findings demonstrate that there is not. In recent years with the increase in tDCS research it has become apparent that the “cathodal impairs and anodal improves” dichotomy is no longer appropriate especially when investigating cognitive performance (Jacobson et al., 2012). Alongside this it is critical to fully understand task you are using, including but not restricted to, the effects of practice, at an observable behavioral level but also at the neural level.

## Author contributions

AE designed the experiments, helped with data collection, advised on analysis and co-wrote the paper. KB helped design the experiments, carried out data collection and analysis and co-wrote the paper. AL helped design the experiments, advised on analysis and co-wrote the paper.

### Conflict of interest statement

The authors declare that the research was conducted in the absence of any commercial or financial relationships that could be construed as a potential conflict of interest.
